# Quorum Sensing and Self-Quorum Quenching in the Intracellular Pathogen *Brucellamelitensis*

**DOI:** 10.1371/journal.pone.0082514

**Published:** 2013-12-11

**Authors:** Matthieu Terwagne, Aurélie Mirabella, Julien Lemaire, Chantal Deschamps, Xavier De Bolle, Jean-Jacques Letesson

**Affiliations:** URBM, Department of Biology, University of Namur, Namur, Belgium; National University, Costa Rica

## Abstract

*Brucella* quorum sensing has been described as an important regulatory system controlling crucial virulence determinants such as the VirB type IV secretion system and the flagellar genes. However, the basis of quorum sensing, namely the production of autoinducers in *Brucella* has been questioned. Here, we report data obtained from the use of a genetic tool allowing the *in situ* detection of long-chain *N*-acyl-homoserine lactones (AHL) activity at single bacterium level in *Brucella melitensis*. These data are consistent with an intrinsic production of AHL by *B. melitensis* in low concentration both during *in vitro* growth and macrophage infection. Moreover, we identified a protein, named AibP, which is homologous to the AHL-acylases of various bacterial species. *In vitro* and during infection, expression of *aibP* coincided with a decrease in endogenous AHL activity within *B. melitensis*, suggesting that AibP could efficiently impair AHL accumulation. Furthermore, we showed that deletion of *aibP* in *B. melitensis* resulted in enhanced *virB* genes expression and VirB8 production as well as in a reduced flagellar genes expression and production of FlgE (hook protein) and FliC (flagellin) *in vitro*. Altogether, these results suggest that AHL-dependent quorum sensing and AHL-quorum quenching coexist in *Brucella*, at least to regulate its virulence.

## Introduction

Quorum sensing (QS), in its broadest sense, is a regulatory system that allows genetic reprogramming in response to small diffusible signalling molecules called autoinducers that are produced and released by bacteria [1]. Once a threshold concentration is reached, autoinducers modulate the activity of a target sensor kinase or a transcriptional regulator, leading to the induction or repression of target genes. This threshold level is usually reached at high bacterial population density, and QS allows individual cells to coordinate gene expression at the level of the whole population in a cell-density-dependent manner [2].However, production of autoinducers by only few bacteria in a restricted environment with limited diffusion rate can be sufficient to reach the threshold concentration [3], and even a single bacterium in an enclosed environment is able to engage QS [4]. Thus QS signal molecules allow bacteria not only to sense quorum *per se*, but also environmental factors such as diffusion and confinement [1]. QS is used by many bacterial species to regulate numerous functions, including virulence, symbiosis, biofilm formation, swarming motility, antibiotic resistance and plasmid conjugation [5], [6].

In Gram-negative bacteria, *N*-acyl homoserine lactones (AHLs) are commonly used as the chemical cues of QS. They are synthesized by LuxI-type AHL-synthases from acyl-acyl carrier proteins and S-adenosylmethionine [7], and they differ one from another on the length of their acyl side chains that additionally may contain C3 substitutions (either a hydroxyl or a carbonyl group). The amphiphilic nature of AHLs allows them to freely diffuse across bacterial membranes, although an efflux pump is required in some cases [8]. Once the threshold intracellular concentration is reached, AHLs bind to their cognate LuxR-type transcriptional regulator(s) and modulate their activity [5], [6].

The Gram-negative bacteria *Brucella* spp. are the etiologic agent of brucellosis, a chronic disease affecting wild and domestic animals but also humans worldwide [9]. Although details on their infectious cycle within natural mammalian hosts are lacking, it is commonly assumed that successful establishment of persistent infections by *Brucella* spp. is linked to its ability to survive and replicate within host phagocytic cells, while remaining inconspicuous and avoiding a strong inflammatory response at the onset of infection [10], [11].

In previous studies, we have reported the identification of QS components in *Brucellamelitensis*. First, we demonstrated *in vitro* a low level of production of AHLs, namely *N*-dodecanoyl-homoserine lactone (C12-HSL) and probably *N*-3-oxo-dodecanoyl-homoserine lactone (3-oxo-C12-HSL), although *Brucella* lacks a classical AHL-synthase [12]. Secondly, we identified two conserved LuxR-type regulators named VjbR and BabR (also known as BlxR) that contain a predicted N-terminal AHL-binding domain and a C-terminal HTH DNA-binding domain [13], [14]. Moreover, several works by independent groups have suggested that **(i)** VjbR and BabR are global transcriptional regulators controlling genes involved in *Brucella* virulence, stress response and metabolism, some of them being identified as direct VjbR targets [13]–[16]; and **(ii)** that C12-HSL modifies the transcriptional activity of VjbR and BabR [12]–[17].

The *virB* operon, encoding proteins of the VirB type IV secretion system (T4SS), and flagellar genes were the first identified QS targets in *Brucella*, and both are induced by VjbR [13]. The T4SS is one of *Brucella*’s major virulence factors, as it is essential for the control of *Brucella*-containing vacuole (BCV) trafficking towards a replication permissive organelle [18]. Expression of the *virB* operon requires VjbR and its deletion causes strong attenuation of *Brucella* in cellular infection models as well as *in vivo* [19], [20]. While non-motile, *Brucella* spp. possess 31 genes encoding flagellar proteins [21]. VjbR is a transcriptional activator of the gene encoding the flagellar master regulator FtcR [22]. Flagellar genes are required for the establishment of chronic infections, as all *B. melitensis* flagellar mutants tested so far, except for the ▵*fliC* and ▵*flbT* strains, are attenuated *in vivo* [20], [21], [23], [24].

The role of *Brucella* QS components has also been evaluated during infection. Deletion of *vjbR* was shown to cause a strong attenuation of *B. melitensis* virulence both in cellular and murine infection models [13]. Conversely, it was proposed that C12-HSL negatively influences *Brucella* virulence as its exogenous addition on early infected macrophages impairs *B. melitensis* intracellular replication, an effect that has been suggested to occur through the alleviation of VjbR-mediated induction of the *virB* operon expression [16]. Thus, VjbR would promote *Brucella* virulence, whereas C12-HSL would dampen it.

Despite these observations, it is still not known whether C12-HSL, 3-oxo-C12-HSL, VjbR and BabR function in a *bona fide* QS regulatory system in *Brucella* and what would be the environmental factor monitored by AHLs. Moreover, as *Brucella* AHL synthase remains elusive [25] and, as only low concentration of AHLs has been detected in culture supernatants, their intrinsic production by *Brucella* was recently questioned [17].

In the current study, we used a genetic tool that provided results consistent with an intrinsic production of long-chain AHLs within *B. melitensis* both in culture *in vitro* and during cellular infection. In both cases, we found that the concentration of QS signals within *B. melitensis* was not population-density-dependent. *In vitro*, this was largely due to the expression of *aibP*, which encodes an AHL-inactivating protein homologous to the AHL-acylases of various bacterial species. Therefore, in addition to be capable of QS, we propose that *Brucella* is also capable of self “quorum quenching” [26]. Similarly to QS, AibP-mediated quorum quenching was found to regulate both *virB* and flagellar genes expression, although it acted on them in opposite ways.

## Results

### *In situ* detection of long-chain AHL activity in *B. melitensis*

*Brucella* has been described to produce long-chain AHLs, namely C12-HSL and probably 3-oxo-C12-HSL. However, they were only detected at low levels in supernatants of *B. melitensis* cultures in stationary phase of growth [12]. We hypothesized that this low extracellular concentration could be due to the control of AHL synthesis by *Brucella* and/or to the limited diffusion of these hydrophobic molecules across bacterial membranes [8]. For these reasons, we investigated the AHL production kinetics of *B. melitensis in vitro* by using a sensitive AHL-responsive reporter system rather than by monitoring and quantifying their appearance in culture medium. The AHL reporter system developed by Hentzer *et al*. [27], which is based on the production of the unstable GFP(ASV) following activation of the *Pseudomonas aeruginosa* LuxR-type regulator LasR (Figure S1A), appeared to be suited for the detection of endogenous AHL activity in *B. melitensis*. Indeed, although 3-oxo-C12-HSL is the cognate signal molecule of LasR, the regulator responds to AHLs with chain length above C10 [27]. Moreover, the instability of GFP(ASV) makes it an interesting reporter protein to quantify rapid changes in AHL concentrations. In order to use it in *Brucella* with a high sensitivity, this genetic tool was cloned on the medium copy and broad host range vector pBBRMCSI (Figure S1A). A control plasmid that does not contain *lasR* was used to ensure that no endogenous regulator could induce *gfp*(ASV) in *Brucella* (Figure S1A).

Prior to its use in *Brucella*, we tested the sensitivity and confirmed the specificity of the QS reporter plasmid in *Escherichia coli*, in which QS is not based on endogenous AHL production [28]. Analysis of GFP(ASV) production has been performed by flow cytometry after a 4h incubation with various concentrations of exogenous synthetic AHLs and fixation of bacteria. As expected, *gfp*(ASV) was not induced in the absence of AHL (Figure S1B). Moreover, the system is specific for long-chain AHLs, as C12-HSL and 3-oxo-C12-HSL, but not C4-HSL triggered a dose-dependent response (Figure S1B). As expected, the cognate signal molecule of LasR, *i.e.* 3-oxo-C12-HSL, was the most efficient inducer of the system (Figure S1B).

**Figure 1:**
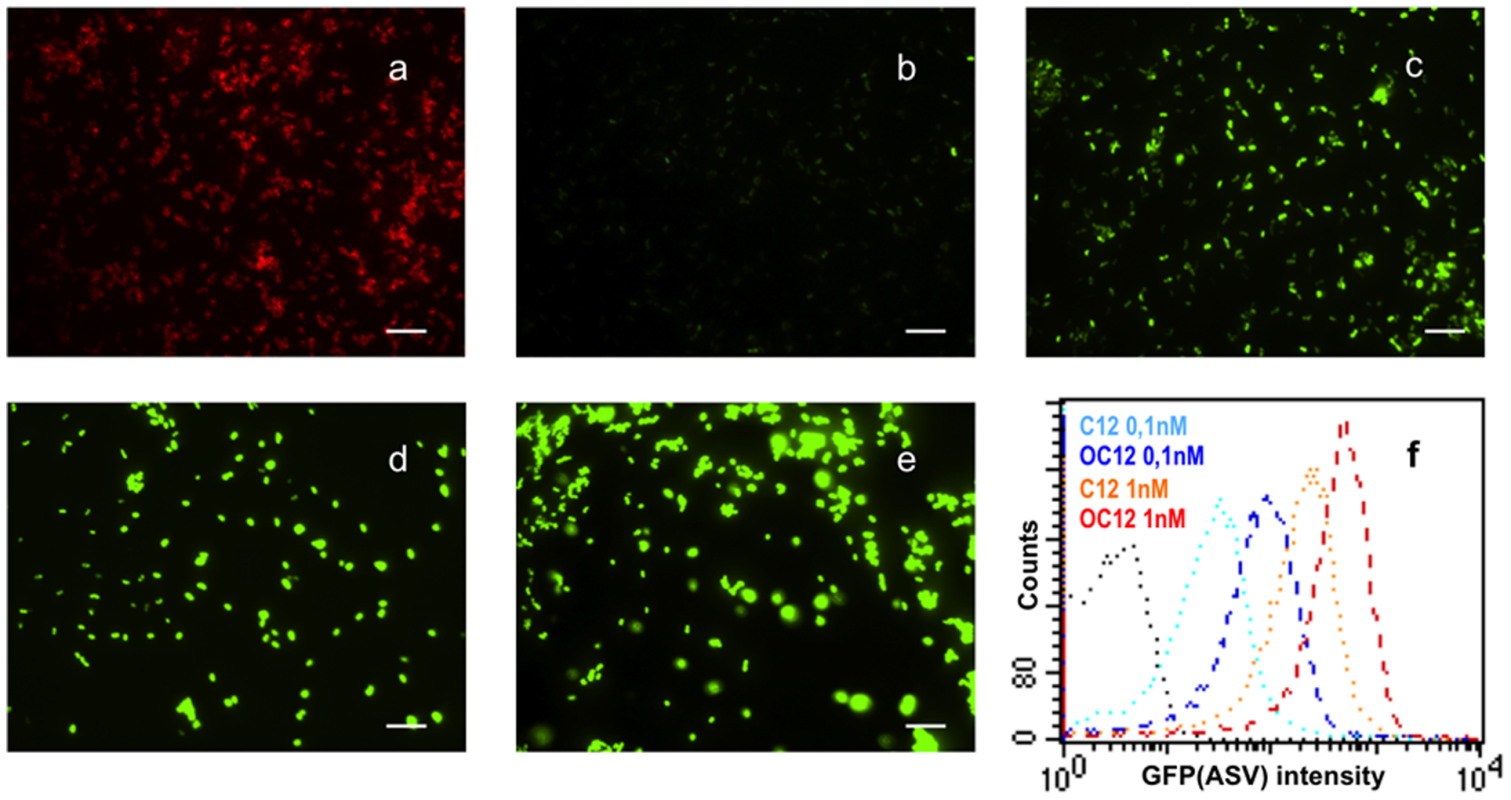
Validation of the specificity and sensitivity of the QS reporter system in *Brucella melitensis* 16M. (**a**) Immunofluorescence of the *B. melitensis* control strain incubated 4 hours with C12-HSL (1 µM) and labelled with monoclonal A76-12G12 anti-LPS antibody (red). No GFP(ASV) signal is detected. (**from b to e**) Observation of GFP(ASV) production by the *B. melitensis* reporter strain after a 4h incubation with various concentrations of synthetic C12-HSL; (**b**) 1nM, (**c**) 10nM, (**d**) 100nM, (**e**) 1 µM; scale bar 5 µm. (**f**) Measurement of GFP(ASV) fluorescence intensity by flow cytometry (5×10^4^ events acquired) in the *B. melitensis* QS reporter strain fixed after a 4h-incubation with 0.1nM or 1nM of C12-HSL or 3-oxo-C12HSL. The *B. melitensis* control strain was used as a negative control (black dotted line). The results are representative of at least two independent experiments.

The QS reporter plasmid and the control plasmid were then separately introduced into *B. melitensis* 16M by conjugation to give rise to *B. melitensis* QS reporter and *B. melitensis* control strains, respectively. Fluorescence microscopy and flow cytometry analysis were used to validate both strains. Similar to what has been observed with *E. coli*, the *B. melitensis* QS reporter strain but not the control strain responded to exogenously supplied C12-HSL and 3-oxo-C12-HSL in a concentration-dependent manner. Indeed, both the number of GFP(ASV)-positive bacteria and the GFP(ASV) fluorescence intensity increased proportionally to signal molecules concentration (Figure 1). The absence of GFP(ASV) signal in the control strain incubated with C12-HSL suggested that no *Brucella* regulator could take place of LasR to induce the reporter gene. The QS reporter vector was thus adapted to our purpose, as activation of heterologously expressed LasR, and the subsequent induction of *gfp*(ASV) reporter gene in *B. melitensis* was expected to reflect the presence of one or several long-chain AHLs within this species.

### Endogenous AHL activity within *B. melitensis* does not increase proportionally to bacterial density

**Figure 2:**
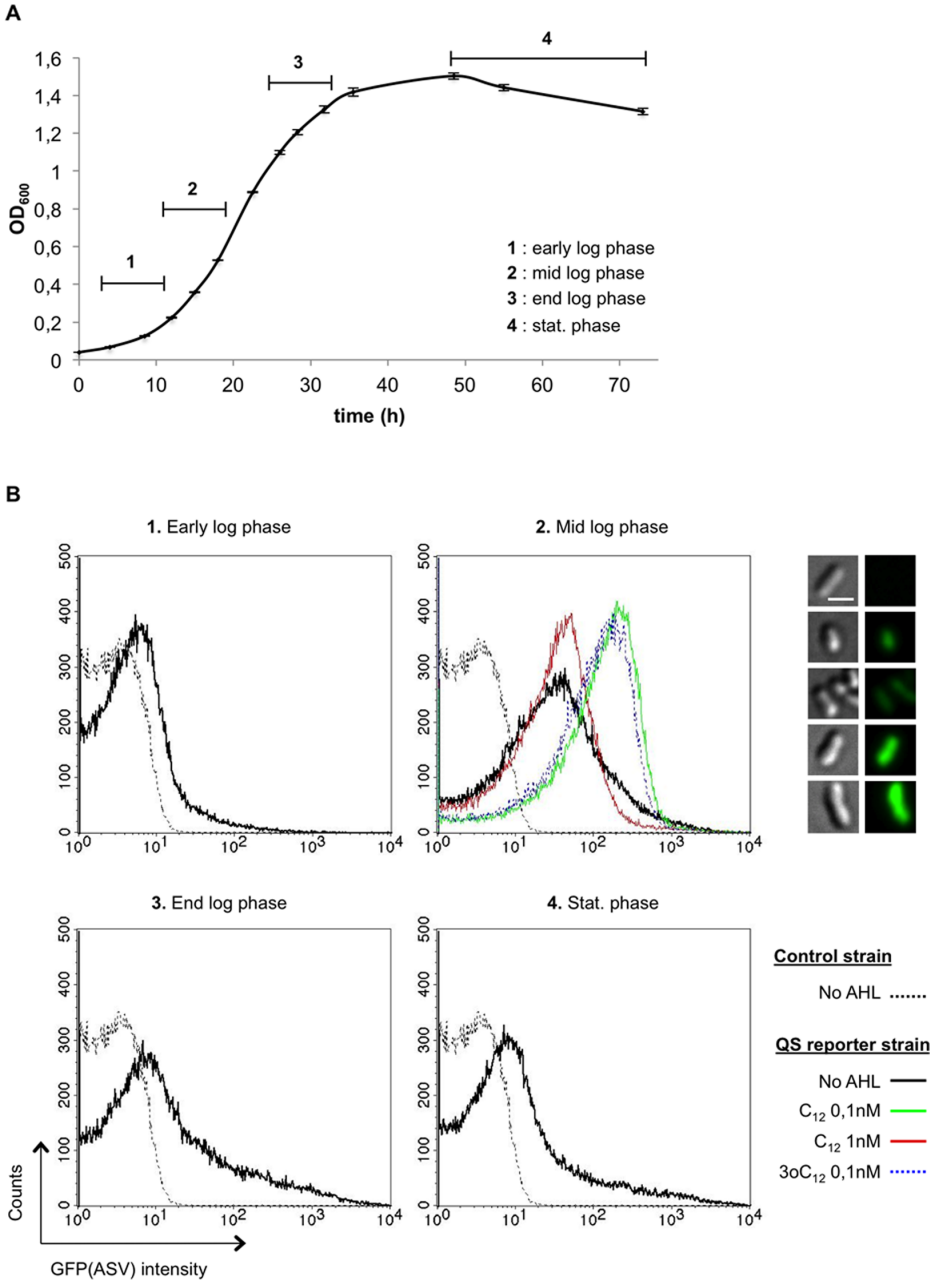
Transient production of long-chain AHLs by *B. melitensis* in liquid culture. The *B. melitensis* QS reporter strain was grown in 2YT and cell density was determined when fluorescence intensity of GFP(ASV) was assessed by flow cytometry (5×10^4^ events acquired). (**A**) Growth curve of the *B. melitensis* QS reporter strain. Numbers represent the 4 distinct phases of growth. OD_600_, optical density at 600nm. (**B**) Histograms of GFP(ASV) fluorescence intensity representative of the growth phases represented in A. The *B. melitensis* control strain was used as a negative control. In (**B2**), the peak of GFP(ASV) fluorescence intensity due to endogenous AHLs was compared with results obtained after a 4h-incubation of the *B. melitensis* QS reporter strain with synthetic C12-HSL or 3-oxo-C12-HSL (bacteria from the early log phase were used). The insets show differential interference contrast (DIC) and FITC fluorescence microscopy of (from top to bottom) the negative control strain, the QS reporter strain in the absence of exogenous AHL and incubated with C12-HSL 0.1nM, with C12-HSL 1nM, and with 3-oxo-C12-HSL 0.1 nM respectively. Results are representative of three independent experiments.

As the LasR-based reporter system allows for semi-quantitative detection of long-chain AHLs activity, we used it in *B. melitensis* 16M to follow its own AHLs production during *in vitro* growth. The *B. melitensis* QS reporter and control strains were grown in 2YT rich medium. At different times post-inoculation, bacterial samples were fixed and analysed by flow cytometry for GFP(ASV) production. Results representative for each of the different 4 growth phases determined by growth rate calculation are presented in Figure 2. GFP(ASV) signal was not observed for the control strain, whatever the growth phase considered. Conversely, we could detect a GFP(ASV) signal at each time of bacterial growth when using the QS reporter strain (Figure 2B). Detecting an endogenous long-chain AHL activity in *B. melitensis* is consistent with the previously reported production of C12-HSL and 3-oxo-C12-HSL *in vitro* [12]. Interestingly, a peak of GFP(ASV) production was observed during the mid-log phase, *i.e.* between 12 and 18h of growth, which corresponded to an OD_600_ between 0.3 and 0.6 (Figure 2A and 2B). It was followed by a decrease at the end exponential and stationary phases at which bacterial density is the highest (Figure 2A and 2B). Moreover, the measurement of GFP(ASV) fluorescence intensity after the addition of synthetic AHLs on the *B. melitensis* QS reporter strain harvested in the early log phase (corresponding to a low intrinsic production of AHL) indicated that maximal intrabacterial concentration of endogenous long-chain AHL in the tested conditions would not be higher than 1nM of C12-HSL or 0.1nM of 3-oxo-C12-HSL (Figure 2B panel 2).

### Identification of a conserved putative AHL-acylase in *Brucella*

We hypothesized that the decrease in intrabacterial AHL activity observed during vegetative growth could reflect a decrease in AHL synthesis, and/or could be due to the degradation of signal molecules. As the mechanisms of AHL synthesis in *Brucella* remains unknown, the first hypothesis could not be further tested. However, in order to determine whether AHL production in *Brucella* could be regulated by self-quorum quenching activity, a bioinformatic screen of *Brucella* spp. genomes was carried out to identify homologs of the two classes of AHL-degrading enzymes described to date, *i.e.* AHL-lactonases and AHL-acylases. No AHL-lactonase homologous to *Bacillus* spp. AiiA [26], *Rhodococcus erythropolis* QsdA [29], or *Ochrobactrum* sp. strain T63 AidH [30] were found. However, BLASTP searches allowed us to find a hypothetical protein in *Brucella* sharing 17–28% identity with characterized or predicted AHL-acylases from various species (e value from 1e-04 to 3e-86) (Figure S2). This 761 aa-long protein was named AibP (AHL-inactivating *Brucella* protein) and was found in all of the sequenced *Brucella* genomes, except for that of *B. ovis*. In *B. melitensis*, a frameshift mutation due to a 1 bp deletion is responsible for the interruption of *aibP*, which then covers two ORFs, *i.e.* BMEII0212 and BMEII0211 (Figure S2). No homologs were found in close phylogenetic relatives of *Brucella*, such as *Ochrobactrum anthropi*, *Sinorhizobium* spp., *Mesorhizobium loti*, *A. tumefaciens*, *Rickettsia* spp. or *Bartonella* spp. As part of the N-terminal nucleophile (Ntn) hydrolases superfamily, AHL-acylases are produced into a single inactive precursor that consists of a signal peptide followed by a α-subunit, a spacer sequence and a ß-subunit [31]–[34]. Further *in silico* analysis of AibP showed that conserved regions are equally observed in the predicted α- and ß-subunits. Unlike most AHL-acylases, *Brucella* spp. homolog is not predicted to have a N-terminal signal peptide. More important is the conservation in AibP of amino acids known to be part of the active site of characterized Ntn hydrolases and that are essential to their post-translational processing and/or their enzymatic activity (Figure S2) [35]–[39]. Among them, the glycine-serine pair, which represents the cleavage site between the spacer peptide and the ß-subunit, generating a free N-terminal nucleophile serine that has been demonstrated to be essential to the activity of *Ralstonia* AHL acylase [31].

### The decrease in AHL activity within *Brucella* is correlated with *aibP* expression

**Figure 3:**
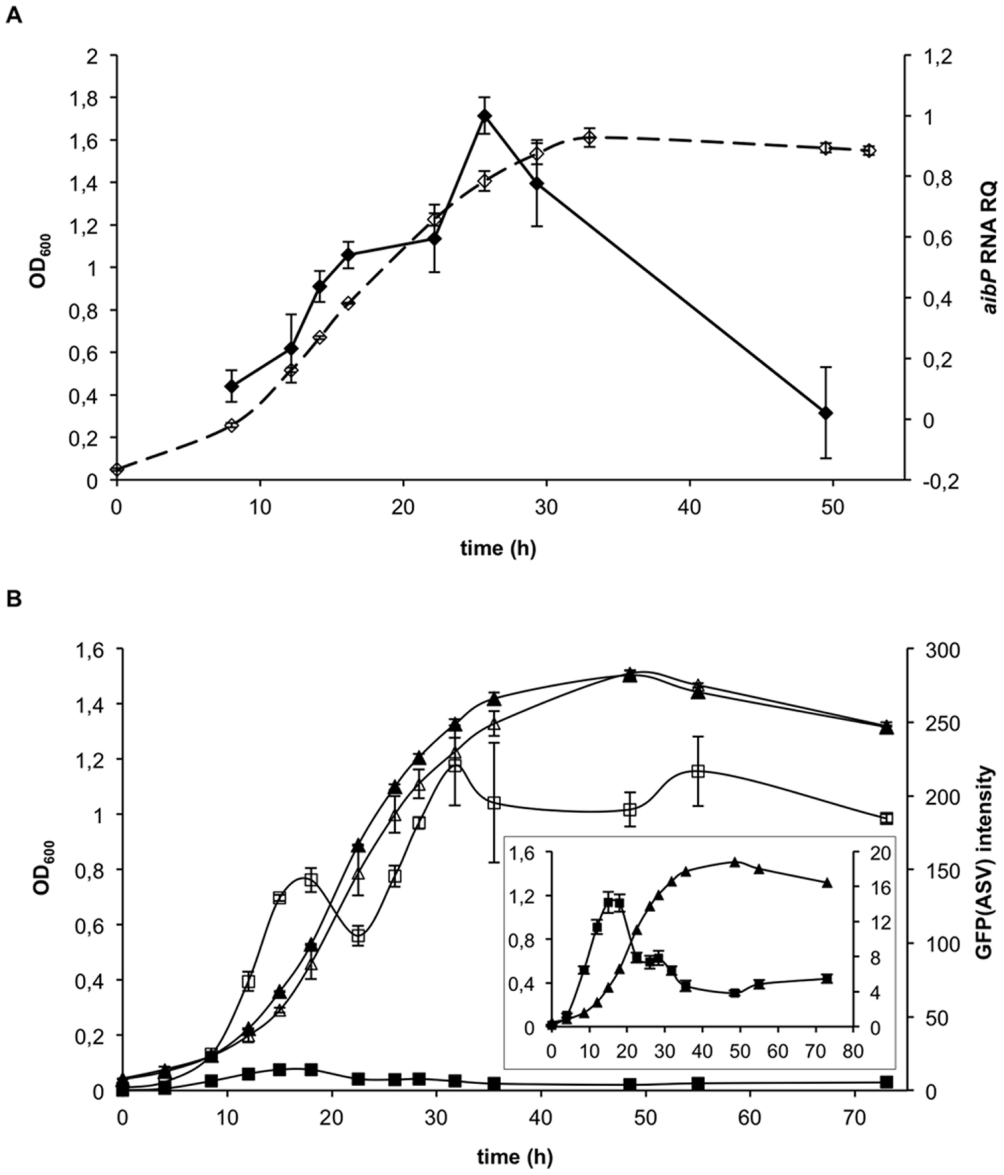
The decrease in *Brucella* AHLs activity during *in vitro* growth is linked to *aibP* expression. (**A**) The relative amount of *aibP* mRNA (black forms) during vegetative growth of *B. melitensis* (open forms) in 2YT medium was determined by qRT-PCR. Result is representative of three independent experiments. Error bars represent standard deviation from biological triplicates. OD_600_, optical density at 600nm; RQ, relative quantity. (**B**) The activity of endogenous long-chain AHLs was followed using the QS reporter system. *B. melitensis* wt (black forms) and ▵*aibP* (open forms) QS reporter strains were grown in 2YT and cell density was determined (triangle) for each culture when GFP(ASV) fluorescence intensity was evaluated (square). The activity of endogenous long-chain AHL(s) at each time is expressed as the geometric mean of GFP(ASV) fluorescence intensity measured by flow cytometry (5×10^4^ events acquired). The *B. melitensis* control strain was used as a negative control (not shown). Insert: the scale of the graph was modified for analysis in the wt strain. Results are representative of three independent experiments. Error bars represent standard deviation from biological triplicates.

In order to know whether we could link the decrease in AHL activity observed when *B. melitensis* 16M entered the end log phase of growth in 2YT with the expression of *aibP*, quantitative real-time PCR (qRT-PCR) was performed to follow variations in the relative level of its transcript. We found that *aibP* mRNA level increased during exponential growth up to ten-fold, and was followed by a decline upon entry into stationary phase (Figure 3A). As the peak of *aibP* mRNA level coincided with the decrease in AHL activity (Figure 2B), it was tempting to speculate that both events are directly linked. In order to further test this hypothesis, we constructed an *aibP* non-polar mutant of *B. melitensis* (▵*aibP*). This mutant produced smooth LPS, and bacterial and colony morphology were not different from the wt strain (data not shown). Moreover, the aggregative phenotype previously reported for some *B. melitensis* QS-mutants [16], [40] was not observed (data not shown). By using the QS reporter plasmid described above, we could first suggest that AibP inactivates synthetic C12-HSL and 3-oxo-C12-HSL supplied to *B. melitensis* cultures, as the mean GFP(ASV) fluorescence intensity measured by flow cytometry after incubation of the wt or the ▵*aibP* strain with these AHL was higher in the mutant than in the wt (Figure S3). More significantly, we could also suggest an activity of AibP toward self-generated AHL. Indeed, by monitoring the endogenous long-chain AHL activity in the ▵*aibP* mutant (*i.e.* in the absence of any added AHL) we found that the mean fluorescence intensity of GFP(ASV) in the mutant strain population was higher than in the isogenic wt strain population throughout all bacterial growth phases (Figure 3B). This suggests that the mutant accumulated higher levels of long-chain AHL during *in vitro* growth: while their concentration decreased during the log phase of *B. melitensis* wt growth, bacteria showed the typical population density-dependent accumulation of AHL in the absence of *aibP* (Figure 3B). This result strongly suggests that AibP is an efficient enzyme with an activity against physiological concentrations of long-chain AHLs produced by *Brucella*, and that it plays a critical role in the regulation of QS signals concentration *in vitro*.

### AHL synthesis and degradation occur within *B. melitensis* during macrophage infection

**Figure 4:**
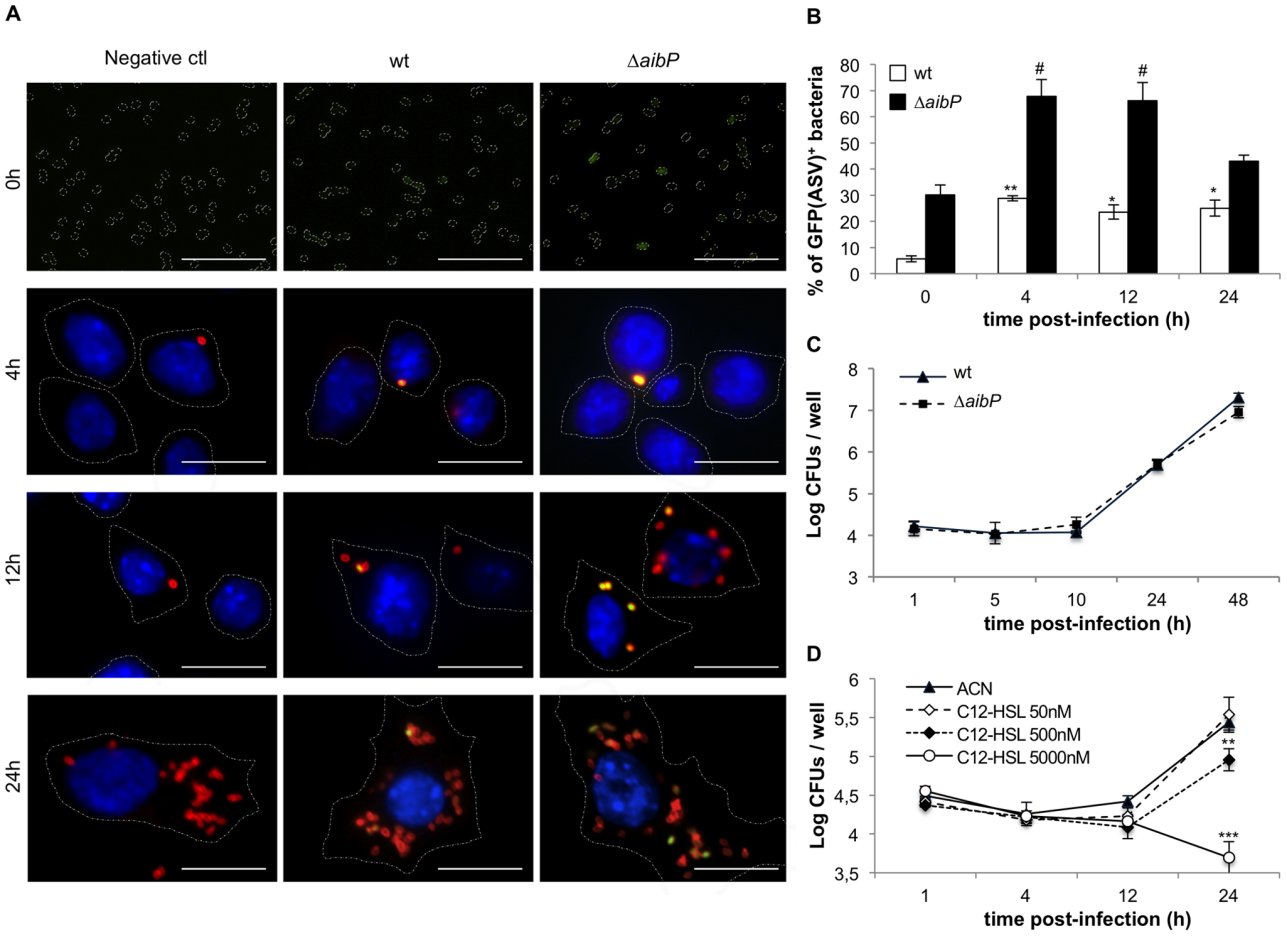
*B. melitensis* both produces and degrades long-chain AHLs during macrophage infection. (**A and B**) *B. melitensis* wt and ▵*aibP* QS reporter strains were used to infect monolayers of RAW264.7 murine macrophages. Prior to (0h) and during infection, bacteria or cells were fixed, bacteria were labelled with a monoclonal A76-12G12 anti-LPS antibody and DNA was labelled with DAPI. (A) Immunofluorescence micrographs are representative from at least two independent experiments. Bacteria LPS appears in red, DNA in blue. Scale bar, 10 µm. (B) The percentage of GFP(ASV)-positive bacteria at different times post-infection was determined as described in the Material and Methods section. Error bars represent the standard deviation from two independent experiments. Data have been analyzed by ANOVA I after testing the homogeneity of variance (Bartlett). * and ** denote significant differences (*P* < 0.05 and *P* < 0.01) in relation to wt bacteria prior to infection (0h) while # denotes a significant difference (*P* < 0.05) in relation to ▵*aibP* bacteria prior to infection. (**C**) Intracellular replication of *B. melitensis* wt and ▵*aibP* strains in RAW264.7 murine macrophages. At indicated times, cells were lysed and intracellular colony forming units (CFUs) were determined. Error bars represent the standard deviation of triplicates in one representative experiment out of three. (**D**) RAW264.7 macrophages were infected with *B. melitensis* wt in the presence of C12-HSL or ACN (negative control) and treated as described (C). ** and *** denote significant (*P* < 0.01 and *P* < 0.001 respectively) differences in relation to infection by wt bacteria in the presence of CAN (Bartlett and ANOVA I analysis).

*Brucella* is usually referred to as a “facultatively extracellular intracellular parasite” [41] to emphasize its impressive ability to survive and replicate in phagocytic cells, which are thought to be the primary niche of the bacteria within the host. We took advantage of the QS reporter tool to determine whether *Brucella* produces AHL during its intracellular life. The murine macrophage line RAW264.7 was infected with *B. melitensis* wt or ▵*aibP* strain each bearing either the control or the QS reporter plasmid and grown to stationary phase in RPMI medium supplemented with erythritol. In this condition, the GFP(ASV) signal of both wt and ▵*aibP* strains was found drastically reduced compared to bacteria grown in 2YT (Figure S4). Having an inoculum containing mostly GFP(ASV)-negative bacteria was important in order to highlight a potential *de novo* synthesis of AHLs during macrophage infection. Prior to infection (0h) as well as at 4-, 12- and 24 hours post-infection (p.i.), cells were fixed and examined by fluorescence microscopy in order to measure the mean intensity of GFP(ASV) fluorescence in individual bacteria (Figure 4A). The number of GFP(ASV)-positive bacteria (Figure 4B) was determined as described in the Material and Methods section. Compared to the inoculum (0h), we observed a reproducible increase (minimum 5-fold) in the number of GFP(ASV)-positive *B. melitensis* wt bacteria 4h p.i., suggesting an induction of AHL production following infection (Figures 4A and 4B). This percentage was not found to increase at later time p.i. even when bacteria entered the replication phase (Figures 4A and 4B). Thus, AHL production by *B. melitensis* did not seem to depend on the bacterial number in infected cells. Similar to what has been observed *in vitro*, both the number of GFP(ASV)-positive bacteria and the fluorescence intensity of GFP(ASV) were higher when the *▵aibP* strain was used to infect RAW 264.7 macrophages (Figures 4A and 4B), indicating that AibP-mediated inactivation of self-produced AHL occurred during cell infection.

It is noteworthy that, despite its apparent inability to control AHL concentration as the wt does, the *▵aibP* mutant strain did not display any defect in its capacity to enter, survive and replicate in various cell lines, as indicated by CFUs recovery (see Figure 4C for RAW264.7 infection; data not shown for bovine SV40 macrophages and HeLa cells). This observation contrasts with the effect of exogenous C12-HSL addition on early infected cell that has been previously reported to impair bacterial replication [16]. However this inhibitory effect was found to occur mostly when very high concentrations of C12-HSL (5 µM) were used (Figure 4D).

### AibP-mediated quorum quenching regulates expression of virulence genes

Since QS has been shown to play a role in the control of virulence genes expression in *Brucella*, we investigated whether its regulation by AHL inactivation through AibP could also impact it. We chose to assess the expression of the *virB* operon and flagellar genes in the ▵*aibP* mutant as these genes were the first identified QS targets required for full virulence of *Brucella*.

**Figure 5:**
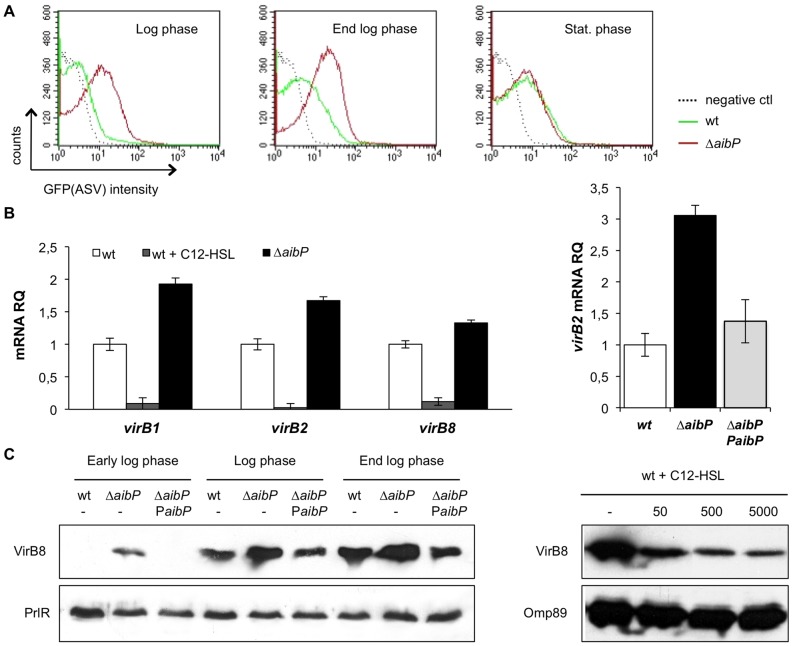
Self-quorum quenching regulates *virB* genes expression. (**A**) *B. melitensis* wt and ▵*aibP* strains both carrying the pBBR *PvirB*-*gfp*(ASV) plasmid were grown in 2YT and GFP(ASV) fluorescence intensity was measured at indicated phases of growth by flow cytometry (5×10^4^ events acquired). Results are representative of two independent experiments. (**B**) The relative abundance of *virB1*, *virB2*, and *virB8* mRNAs was determined by qRT-PCR on RNA isolated from bacteria harvested at the early exponential phase of growth in 2YT supplemented or not with exogenous C12-HSL (5 µM). Deletion of *aibP* results in significant upregulation of *virB* genes (P<0.001 in Student’s t test), whereas exogenous C12-HSL significantly downregulates their expression (P<0.001 in Student’s t test). ▵*aibP PaibP* on the right panel is the complemented strain. Results are representative of two independent experiments. Error bars represent standard deviation from biological triplicates. (**C**) (Left panel) Western Blot analysis of VirB8 production performed on whole protein lysates of bacteria harvested at the indicated phases of growth in 2YT. ▵*aibP PaibP* is the complemented strain. (Right panel) Bacteria were harvested in the log phase of growth in 2YT in the absence (–) or in the presence of C12-HSL. The VirB8 protein was detected at its expected size (26,5 kDa). Detection of PrlR or Omp89 proteins was used to normalize total protein content.

First, we compared the activity of the *virB* operon promoter (P*virB*) in the wt and ▵*aibP* strains during their growth in 2YT medium. A fusion of the *B. melitensis virB1* upstream region to the promoterless *gfp*(ASV) was constructed and the resulting plasmid pBBR-P*virB*-*gfp*(ASV) was introduced into both strains. Bacteria carrying the pBBR-P*virB*-*gfp*(ASV) vector were fixed at different times of the growth curve and GFP(ASV) production was measured by flow cytometry. Results representative of each growth phase are shown in Figure 5A. In agreement with the previously reported growth phase-dependent regulation of *virB* operon expression in rich medium [13], [42], we noted that P*virB* was barely active in *B. melitensis* wt strain during the exponential growth, while it was induced at the end log phase and reached its maximal activity in the stationary phase (Figure 5A). Interestingly, an earlier induction of P*virB* in the log phase was observed for the ▵*aibP* mutant (Figure 5A). Moreover, P*virB* activity at the end log phase of growth remained higher in the mutant, while it returned to the wt level in the stationary phase (Figure 5A). The earlier induction of *virB* operon in the ▵*aibP* mutant was confirmed at the mRNA and protein levels respectively by qRT-PCR on *virB1*, *virB2* and *virB8* mRNAs (Figure 5B, left panel), and by Western blot analysis of VirB8 production in cultured bacteria (Figure 5C). Overexpression of *virB*2 (Figure 5B, right panel) and overproduction of VirB8 (Figure 5C) were both compensated by the expression of *aibP* from a replicative vector, consistent with complementation of the *aibP* deletion. This phenotype of the ▵*aibP* strain was in striking contrast with the downregulation of *virB* genes expression and VirB8 production in wt *B. melitensis* grown in the presence of C12-HSL (Figures 5B and 5C). However, the absence of a negative effect of long-chain AHL accumulation in the ▵*aibP* strain is in agreement with its ability to replicate at the wt level intracellularly (Figure 4C).

**Figure 6:**
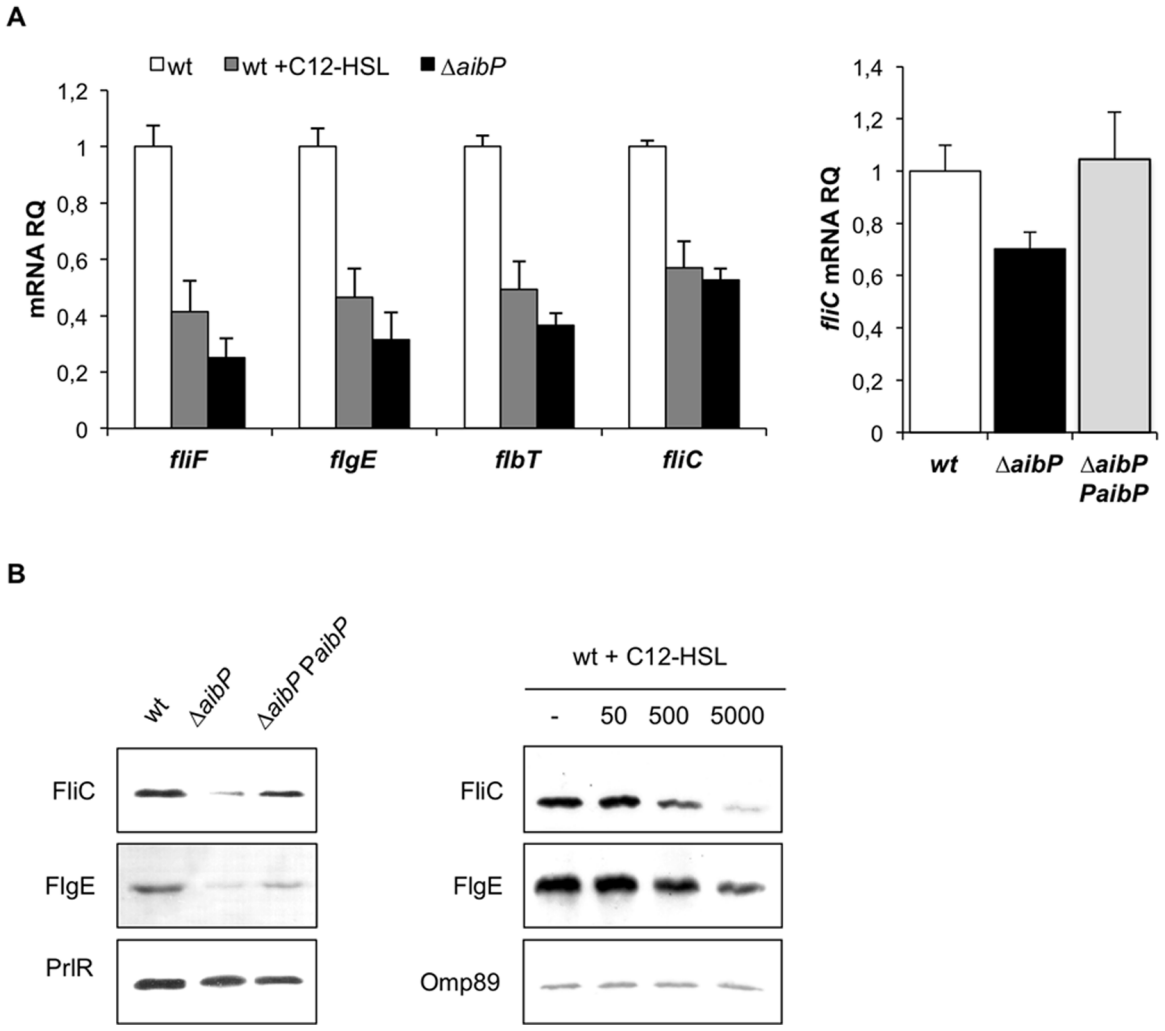
Self-quorum quenching regulates flagellar genes expression. (**A**) The relative abundance of *fliF*, *flgE*, *flbT*, and *fliC* mRNAs was determined by qRT-PCR on RNA isolated from bacteria harvested at the early exponential growth phase in 2YT supplemented or not with exogenous C12-HSL (5 µM). Deletion of *aibP* and addition of exogenous C12-HSL on *B. melitensis* both result in significant downregulation of the expression of the tested genes (P<0.001 in Student’s t test). ▵*aibP PaibP* (right panel) is the complemented strain. Results are representative of two independent experiments. Error bars represent standard deviation from biological triplicates. (**B**) (Left panel) Western Blot analysis of FliC and FlgE production performed on whole protein lysates of bacteria harvested at the early log phase of growth in 2YT. ▵*aibP PaibP* is the complemented strain. (Right panel) Bacteria were harvested at the early log phase of growth in 2YT in the absence (–) or in the presence of C12-HSL. The FliC and FlgE proteins were detected at their expected size (29 kDa and 41 kDa respectively). Detection of the PrlR or Omp89 was used to normalize total protein content.

Flagellar genes are also targeted by QS regulation in *Brucella* [13], [22]. Expression of flagellar genes recently reported as class II and class III genes in the non-classical hierarchy of flagellum biogenesis in *B. melitensis* was monitored by qRT-PCR on bacteria harvested at the early exponential growth phase in 2YT medium. *fliC* is a class III gene that codes for the filament flagellin subunit; *fliF*, *flgE* and *flbT* are class II genes encoding the basal body MS-ring protein, the hook protein and a regulator needed for flagellin production, respectively [43]. Expression of all these genes was significantly downregulated in the ▵*aibP* mutant, and this effect was also observed in wt bacteria supplied with exogenous C12-HSL (Figure 6A). These results were confirmed by Western blot analysis of FlgE and FliC abundance in the wt and ▵*aibP* mutant strains as we observed that both hook protein and flagellin levels were reduced in the ▵*aibP* mutant or when wt was grown in 2YT supplemented with high concentrations of C12-HSL (Figure 6B). Moreover, the use of a replicative plasmid carrying the *aibP* gene allowed complementation of this phenotype (Figure 6A and 6B).

## Discussion

Recent studies have identified and characterized components of a QS system in *B. melitensis*. These include C12-HSL and probably 3-oxo-C12-HSL as signal molecules [12], and VjbR and BabR/BlxR as responding LuxR-type regulators [13]–[16], [44]. However, the fact that *Brucella* lacks a classical AHL-synthase and that only low levels of AHLs were detected in the supernatant of *B. melitensis* stationary-phase cultures brought up questions about the basis of such a system in *Brucella*, namely the intrinsic production of QS cues. Here, we sought to confirm endogenous production of AHLs in *Brucella* by using a sensitive reporter system that allows specific *in situ* detection of long-chain AHLs activity at single bacterium level. In this genetic tool, the *gfp*(ASV) gene is transcriptionally controlled by the *P. aeruginosa* LuxR-type LasR (Figure S1A). Detecting the activation of heterologously expressed LasR within *Brucella* appeared to be suited for our purpose as C12-HSL and 3-oxo-C12-HSL are both potent activator of LasR (Figures S1B and Figure 1). Moreover, this system monitors AHL concentration directly within bacteria, allowing us to bypass the problem of slow diffusion of long chain AHLs across bacterial membrane. Finally, determining the amount of AHLs retained by bacteria rather than extracellular AHLs is particularly relevant as the former can be considered as the level of biologically active QS signals. In this respect, significant differences between the intracellular and extracellular pools of AHLs have been already observed in bacterial cultures [8], [45]. By using this QS reporter system, we observed a production of GFP(ASV) by *B. melitensis* during *in vitro* growth (Figure 2B) but also during its intracellular trafficking within infected macrophages (Figures 4A and 4B). These findings constitute the second data consistent with an intrinsic production of long-chain AHLs by *Brucella* in culture, and suggest for the first time that signal molecules are produced by *B. melitensis* in the relevant context of cell infection.

In several Proteobacteria, the QS system is apparently incomplete. For example, LuxR-type QS regulators that lack a cognate LuxI-like AHL synthase have been called “LuxR orphans” [46] or “solos” [47]. In some AHL-producing Proteobacteria, they could function as “extra” LuxR receptors that are connected to complete AHL-dependent QS systems [48]–[50]. However “solos” have also been found in non-AHL-producing bacteria where they detect AHLs produced by neighbouring bacteria [28], exogenous signals produced by eukaryotic hosts [51] or endogenously produced signalling molecules of different nature [52]. While we cannot rule out the possibility of a response of VjbR and BabR to host-derived molecules or to endogenous signals that are not AHLs, we report here that *B. melitensis* 16M produces signals that activate LasR and that can be inactivated by a protein homologous to AHL-acylases, therefore supporting the idea of an endogenous production of AHLs. The finding that mutations of VjbR in critical residues involved in AHL binding affect *virB* expression in the absence of exogenously supplied AHLs *in vitro* reinforces this idea [16]. It should be noted that the absence of an obvious classical AHL-synthase in a bacterial strain is not synonymous of absence of self-produced QS cues. As for *Brucella*, a *Shewanella* isolate was shown to produce AHLs whereas attempts to identify AHL-synthase(s) were unsuccessful, suggesting a novel family of AHL synthases that remain to be discovered [53].

QS was originally described as used by bacteria to coordinate gene expression at the population level and, thus, as a mean for bacteria to monitor population density [5], [6]. *In vitro*, we found that the GFP(ASV) fluorescence intensity measured within the *B. melitensis* QS reporter strain decreased at the end exponential and stationary phases of growth at which bacterial density is the highest (Figure 2). The QS circuitry in cultured *Brucella* is thus non-classical in that the intrabacterial AHLs concentration does not reflect the cell population density. We found that this was largely due to the expression of *aibP*, which encodes an AHL-inactivating protein homologous to the AHL-acylases of various bacterial species (Figure S2) [31]–[34], [54]–[57]. Indeed, when *B. melitensis* lacks *aibP*, accumulation of GFP(ASV) was observed throughout the growth of the QS reporter strain, suggesting that its deletion restores the typical population density-dependent accumulation of AHLs (Figure 3B).

The identification of a mechanism of signal molecules turnover in *B*. *melitensis* is an additional finding consistent with the hypothesis of a self-AHL production. Along with the production of AHL-antagonists and the enzymatic modification of AHLs, their degradation by AHL-lactonases or AHL-acylases is one of the processes of QS interference known as quorum quenching [26], [58], [59]. AHL-acylases are part of the N-terminal nucleophile (Ntn) hydrolases superfamily comprising the penicillin acylase family enzymes, cephalosporin acylases (CA) and aculeacin A acylases [31]. They hydrolyze the amide bond between the homoserine lactone (HSL) and the acyl-side chain of AHL via a nucleophilic attack, resulting in HSL and fatty acid products that cannot spontaneously reform a functional QS signal molecule [31], [60]. Although the identification of the products released upon AibP-mediated inactivation of AHLs would be required to clearly demonstrate it, the conservation of the Glyα, Ser1ß, His23ß, Tyr33ß and Asn244/269ß residues that are essential for the enzymatic activity of Ntn hydrolases suggests that AibP is an AHL-acylase (Figure S2).

Expression of *aibP* during the exponential growth phase could be the main reason why Taminiau et al. [12] found only low levels of C12-HSL and 3-oxo-C12-HSL in *B. melitensis* stationary-phase culture supernatants. However, our semi-quantitative analysis suggests that even in the absence of AibP, the concentration of AHLs would remain low within *B. melitensis* when compared with other bacteria. Indeed, at the peak of GFP(ASV) fluorescence intensity *in vitro*, we suggest that concentrations of C12-HSL and 3-oxo-C12-HSL within *B. melitensis* wt would not be higher than 1nM and 0.1nM, respectively (Figure 2B panel 2), while they would be approximately ten-fold higher in the ▵*aibP* mutant strain (Figure 3B). In contrast, AHLs reach micromolar concentrations in cultures fluids of bacteria such as *Vibrio fischeri* [61], *A. tumefaciens* [62] and *P. aeruginosa* [63]. A low concentration of endogenous long-chain AHLs and their possible intrabacterial sequestration due to their slow diffusion across membranes could account for our inability to detect AHL production by *B. melitensis* ▵*aibP* in cross-streak assays with biosensors strains, or by incubating the latter with culture supernatants of the former strain (data not shown).

A signal functions as a signal *per se* only if it appears but also disappears at a certain time. This also holds true for QS signals. The inactivation of self-produced AHLs by Gram-negative bacteria could be a regulatory mechanism by which they exit a quorum-sensing mode. Nevertheless, it remains unclear whether it takes place in other bacteria, as AHL-acylases from *P. aeruginosa* (PvdQ) and *P. syringae* (HacA and HacB) and an AHL-lactonase (AttM/BlcC) from *A. tumefaciens* have been suggested to be part of AHL-independent metabolic pathways by acting against endogenous substrates other than self-produced AHLs [32], [33], [45], [64], [65]. Although we cannot yet say for certain that AHLs are the natural substrates of *Brucella* AibP, several observations suggest that it mediates self-quorum quenching. First, the timing of *aibP* expression during *in vitro* growth is consistent with the kinetics of GFP(ASV) production in the *B. melitensis* QS reporter strain, as the peak of *aibP* mRNA level coincided with the decrease in GFP(ASV) intensity (Figure 3). Secondly, on the contrary to *P. aeruginosa* [32], *P. syringae* [33] and *A. tumefaciens* [45], in which deleting genes encoding AHL-inactivating enzymes does not affect the intracellular levels of AHLs, the higher GFP(ASV) fluorescence intensity of the ▵*aibP* strain compared to the wt strain (Figures 3B and 4B) suggests that endogenous expression of *aibP* in *Brucella* would allow degradation of self-produced AHLs and maintain them at low concentrations. Finally, the alterations in the ▵*aibP* mutant of *virB* and flagellar genes expression (Figures 5 and 6), which were the first identified targets of VjbR and BabR [13], [44] support the hypothesis that AHLs inactivation by AibP is effectively capable of quenching QS-dependent functions in *B. melitensis*.

Since its discovery in *Vibrio fischeri* [66], the conceptual role of QS has evolved, notably with the highlighting that bacteria can use autoinducers not only to sense quorum *per se* but potentially any environmental and biotic factor influencing their concentrations (for reviews, see [1], [3], [67]). By using the *B. melitensis* QS reporter strain, we could show an early production of AHLs during cell infection (1h, 4h and 8h p.i.), *i.e.* at a time at which infected macrophages contain only few bacteria (from 1 to 3 in average) that have not yet started replication. This suggests that a single *Brucella* individually enclosed in their vacuole could switch from a “non-quorate” to a “quorate” state, in the absence of neighbouring bacteria. Studies of single *Staphylococcus aureus* entrapped within an endosomal compartment in endothelial cells [68] or within a physical and chemical isolated nanostructure droplet [4] have demonstrated that confinement of an individual isolated bacterium can induce QS. In the case of *Brucella*, VjbR is required early in host cell infection, at least to induce *virB* whose expression reaches a peak at 5h p.i. in *B. abortus* during infection of J774 macrophages [69]. Thus, the expression of a QS regulator along with the production of QS cues at very early times p.i. provide single vacuole-enclosed *Brucella* with the possibility to engage QS. This could result in genetic reprogramming required for virulence genes expression but also for the metabolic adaptation of the bacteria to the host environment [14], [15], [70]. Production of QS cues could thus allow *Brucella* to sense confinement in the BCV rather than quorum.

It is interesting to note that *aibP* is part of the VjbR and C12-HSL regulons [15]. More precisely, it is induced by VjbR and repressed by the addition of exogenous C12-HSL, at least through a VjbR-independent mechanism, which could therefore involve BabR. *aibP* is thus part of the QS network of *Brucella*. Further supporting this idea is our finding that deletion of *aibP* in *B. melitensis* was found to impact the expression of QS-regulated genes (Figures 5 and 6). Interestingly, supplying *B. melitensis* wt cultures with C12-HSL or 3-oxo-C12-HSL (500nM or 5 µM) mimics the effect of *aibP* deletion on flagellar genes expression but not on *virB* expression, although increased concentrations of both AHLs are expected in the ▵*aibP* mutant. A similar observation was made in infected macrophages where the ▵*aibP* mutant replicated at the wt level, whereas addition of 5 µM C12-HSL or 3-oxo-C12-HSL on cells reduced the intracellular replication of *B. melitensis* (Figures 4C and 4D). This suggests that *Brucella* QS is far from being completely understood and that regulation of the *virB* operon is even more complex than anticipated. Since the identification of C12-HSL as the major signal molecule produced by *B. melitensis* [12], studies on *Brucella* QS were based on the use of synthetic C12-HSL either on (i) recombinant QS regulator VjbR [17], (ii) on bacteria during vegetative growth [12], [13] or (iii) on intracellular bacteria at different stages of macrophages infection [16]. They all conclude that VjbR functions as a transcriptional activator of *virB* only in the absence of C12-HSL in order to promote cellular infection. However, it should be noted that the concentration of synthetic signal molecules used to report an effect on VirB (and flagellar proteins) production and bacterial multiplication within cells were at least 500 fold higher than the concentration of endogenous C12-HSL or 3-oxo-C12-HSL estimated in the *B. melitensis* ▵*aibP* mutant (5 µM vs 10 nM). Here, we did not find *B. melitensis* to produce such concentrations of long chain AHLs, which could therefore be physiologically irrelevant. It is also noteworthy that the absence of AibP in *B. melitensis* could result not only in higher C12-HSL and 3-oxo-C12-HSL concentrations but also of other unidentified AHL(s) that would not have been detected by Taminiau et al. [12] because of the previously unsuspected AibP activity. These unidentified AHL(s) could have a positive effect on *virB* expression that would be consistent with its earlier induction in the ▵*aibP* mutant (Figure 5). Moreover, it is possible that AibP impacts *Brucella* virulence not only by regulating the AHLs level, but also by its involvement in yet-to-be discovered metabolic processes. As mentioned earlier, other substrates than AHLs have been either identified or suspected for several so-called quorum-quenching enzymes [65], [71]. The links between virulence and metabolism in *Brucella* become more and more appreciated, and it is now clear that regulatory mechanisms of *Brucella* virulence also regulates metabolism, which in turn impacts bacterial virulence [70]. Thus, if the deletion of *aibP* leads to changes in the metabolic state of *Brucella*, through the ability of AibP to modify (an) unknown substrate(s), this could indirectly result in alteration of *Brucella* virulence.

In conclusion we propose that, although its AHL synthase remains elusive, *B. melitensis* synthesizes low levels of long-chain AHLs both during *in vitro* growth and cell infection, and that it carefully controls their concentration through the expression of *aibP* that encodes an AHL inactivating protein. AHL-dependent quorum-sensing and quorum-quenching would thus coexist, and they both regulate expression of virulence genes, including the *virB* operon and flagellar genes.

## Materials and Methods

### Bacteria and growth conditions

The bacterial strains used in this study were *E. coli* DH10B (Gibco BRL), *E. coli* S17-1 [72] and smooth virulent *B. melitensis* 16M (Biotype1, ATCC 23456). *E. coli* and *B. melitensis* strains were grown with shaking at 37°C, respectively in Luria-Bertani and in 2YT medium (10% yeast extract, 10 g liter^−1^ tryptone, 5 g liter^−1^ NaCl) containing appropriate antibiotics. The *B. melitensis* QS reporter strains used for cellular infection were grown in RMPI 1640 (Gibco) supplemented with 2 g liter^−1^ erythritol.

Chloramphenicol, kanamycin and nalidixic acid were used at 20 µg/ml, 50 µg/ml and 25 µg/ml, respectively.

Synthetic C4-HSL, C12-HSL, and 3-oxo-C12-HSL were purchased from Sigma-Aldrich and prepared in acetonitrile (ACN). They were added to bacterial growth media at the indicated final concentrations. The same volume of ACN was used as a negative control.

*B. melitensis* growth curve in 2YT were performed as follows: a stationary-phase overnight culture (2YT, 10 ml) was back-diluted to obtain an optical density at 600nm (OD_600_) of 0.05. Growth was measured by reading OD_600_ at indicated times post-inoculation. 100-ml cultures were used to follow GFP(ASV) production from *B. melitensis* QS reporter strains and from *B. melitensis* harbouring the pBBR-P*virB-gfp*(ASV) vector. 100-ml or 250-ml cultures were used to harvest bacteria for qRT-PCR analysis. 50-ml cultures were used to harvest bacteria for Western blot analysis. The growth rate constant µ was determined from the following equation: ln N_t_ – ln N_0_  =  µ(t – t_0_) in which N is OD_600_.

### Molecular techniques and plasmids

DNA manipulations were performed according to standard techniques [73]. Primers used are listed in Table 1.

**Table 1:** Primers used in this work.

Primer	Sequence
FPaibP	GGGGTACCCCTTCCGAAATGGTTGGAAGG
RPaibP	GGAATTCCGAAGATCTTCTCCGATATAAGAATGGCCG
FTaibP	GAAGATCTTCGGAATTCCACACAGAAATCGGGGAGG
RTaibP	CGGGATCCCGCAACGTGTCGAGAAACGC
F-BHI*aibP*	CGGGATCCATGAACGTCGCGAGTGC
R-XbaI*aibP*	GCTCTAGATTAAGATGGCTGCATAATCAGG
gfpasv-F	CTCGAGATGCGTAAAGGAGAAGAAC
gfpasv-R	GGTACCTTAAACTGATGCAGCGTAG
pvirB-F	GGATCCGAAGTCCTTTCCGTCCTG
pvirb-R	CTCGAGGTCTCCTTCTCAGAGAATG
aibP-F	AACAATTGGGCGGTGGA
aibP-R	AATTTCATAGGCCCGATGC
flbT-F	AACTTCTGAACGATGCGACAT
flbT-R	AAAGCTGGCGCAGCG
flgE-F	TTCCGTGAACGCTGC
flgE-R	GAAACGAGATCGCCCGT
fliC-F	CTTCGTACAATCGTTCCGGT
fliC-R	CCATGGTCTTCGCATCAGT
fliF -F	CCTACGAGACGCTCTATGTCG
fliF-R	AAGGGAATGCCAGCTTCAC
virB1-F	ACGACAGCACAGTCACTGGAAG
virB1-R	TTCGGCAGATTGTACCTGTTGA
virB2-F	GCAAAAAGTGCTGGACTTGCTA
virB2-R	CCATCTTGTAACCGGACCAGAT
virB8-F	TGGATAAATACTGGCTCTCGCA
virB8-R	GGTTTCGTAGTCCTTTTGCAGC
16S rRNA-F	ACGCCGTAAACGATGAATGTT
16S rRNA-R	CCCAGGCGGAATGTTTAATG

To obtain an AHL-monitor vector usable in *Brucella*, the previously described pMHLAS vector [27] was digested with NotI and the restriction fragment ends were filled in with Klenow DNA polymerase. The fragment containing the P*lac*-*lasR* and P*lasB*-*gfp*(ASV) was then inserted into the EcorV site of pBBR1 MCS-I [74] to give rise to the QS reporter plasmid used in this study (Figure S1A). The control vector was obtained by digestion of the previously described pMHLB [27] with BamHI and HindIII, and insertion of the fragment containing P*lasB*-*gfp*(ASV) into the corresponding sites of the pBBR1 MCS-I [74] (Figure S1A).

The *B. melitensis* 16M ▵*aibP* mutant was obtained by allelic replacement as previously described [75]. Briefly, upstream and downstream regions flanking the BMEII0212-BMEII0211 sequence were amplified by polymerase chain reaction (PCR) from *B. melitensis* 16M genomic DNA using the primer pairs (i) FPaibP and RPaibP, (ii) FTaibP and RTaibP. A second PCR was used to ligate the two PCR products by cohesive ends. The PCR product (*aibP* upstream/downstream) was inserted into the *Asp*718 and *BamH*I sites of pSKoriT*cat* to generate pSKoriT*cat*-▵*aibP*. The *aphA4* non-polar deletion cassette [75] was excised from pUC4*aphA4* with BamHI, and subsequently cloned into the *Bgl*II site of pSKoriT*cat*-▵*aibP* to generate the pSKoriT*Cat*-▵*aibP*::*aphA4* plasmid. The plasmid was transformed into *E. coli* strain S17-1 and introduced into *B. melitensis* 16M by conjugation. Clones for which a double recombination event occurred (Cm^s^, Kan^r^) were selected. Gene replacement was confirmed by PCR.

In order to obtain the complementation vector pBBR1-*aibP*, the BMEII0212-BMEII0211 sequence was first amplified by PCR from *B. melitensis* 16M genomic DNA using the F-BHI*aibP* and R-XbaI*aibP* primers. The PCR product (*BamHI-aibP-XbaI*) was then cloned into the *EcorV* site of pGEM. In a last step, this fragment was excised using BamHI and XbaI, and inserted into the corresponding site of pBBR1 MCS-I [74] downstream the endogenous P*lac*. This final vector was transformed into *E. coli* S17-1 and introduced into *B. melitensis* 16M ▵*aibP* by conjugation.

The pBBR1-*gfp*(ASV) vector used to clone P*virB* upstream the promoterless *gfp*(ASV) was generated as follows: the *gfp*(ASV) gene encoding an unstable variant of GFPmut3 was amplified by PCR from the pMHLAS vector [27] using the gfpasv-F and gfpasv-R primers, and the PCR product (*Xho*I-*gfp*(ASV)-*Asp*718) was inserted into the corresponding site of pBBR1-MCSI vector [74] in the opposite orientation to the P*lac*. The promoter of the *virB* operon was supposed to localize within a sequence that comprises the last 30bp of its upstream ORF and going until their predicted ribosome-binding site (RBS). It was amplified by PCR using the pvirB-F and pvirB-R primers. It was then cloned into the BamHI and XhoI sites of the pBBR1-*gfp*(ASV) vector in order to position the predicted RBS at -6 from the *gfp*(ASV) start codon.

### RNA preparation

For analysis of the relative abundance of *aibP*, flagellar genes and *virB* genes, *B. melitensis* strains were grown as described above. Total RNA was extracted from *B. melitensis* strains as follows. At indicated phases of growth, culture samples (10 ml) were harvested, bacteria were resuspended in 100 µl SDS 10% and 20 µl proteinase K (20 mg ml^−1^) and incubated at 37°C with shaking for 1h. TriPure Isolation Reagent (Roche) (1 ml) was added and suspensions were vigorously shaken. After 10 min of incubation at 65°C, chloroform (300 µl) was added, the suspensions were shaken and incubated at room temperature for 5-10 min. Samples were centrifuged at 14 000 rpm for 15 min at 4°C. Then 500 µl of isopropanol was added and RNA was precipitated overnight at –20°C. After a centrifugation (13 000rpm, 30 min), the pellet was washed with ethanol 75%. RNA was dried at room temperature, resuspended in RNase-free water, and contaminating DNA was removed by DNase I treatment (Fermentas) following the manufacturer’s instructions. RNA samples were assessed for quality by the Agilent Bioanalyzer or by electrophoresis. RNA quantity was measured using a NanoDrop spectrophotometer (ND-1000, Thermo Fisher Scientific).

### Relative quantification of mRNAs via the comparative cycle threshold method

1 µg of total RNA was reverse-transcribed into cDNA by using random hexamer primers and the Transcriptor First Strand cDNA synthesis kit (Roche) following manufacturer’s instructions. 250 ng of cDNA were then used as template for quantitative real-time PCR (qRT-PCR) in 96-well optical reaction plates using Power SYBR® Green PCR Master Mix (12.5 µl, Applied Biosystems) and primers (1 µl and 10 µM each, sequences are listed in Table 1) that were designed with PrimerExpress™ 2.0 (Applied Biosystems) and that allowed amplification of sequences from 80 to 100 bp long. Deionized RNase free water was added to make the total volume up to 25 µl/well. We used the thermal cycling conditions recommended by Applied Biosystems, which included 10min at 95°C for thermal activation of the AmpliTaq Gold ® DNA polymerase and 40 two-step cycles of denaturation for 15s at 95°C and annealing/extension for 1min at 60°C. Technical triplicates were performed. Relative quantification using the ▵▵Ct method was performed for each set of primer in an Applied Biosystems real-time PCR instrument. The samples from wt bacteria were used as a reference, and the 16S rRNA was used for normalization. A negative control used for each qRT-PCR reaction showed that no genomic DNA contamination occurred in the RNA samples (data not shown). Standard deviation was calculated on biological triplicates.

### Western blot analysis

For analysis of FlgE, FliC and VirB8 production, *B. melitensis* strains were grown as described above. At indicated growth phases, bacterial samples were harvested. Bacteria were concentrated in PBS at an OD_600_ of 10 in 50 µl, prior to a 1h-inactivation at 80°C, and addition of 2x SDS-sample buffer. The proteins were resolved on a 12% polyacrylamide gel and transferred to Hybond ECL nitrocellulose membranes (Amersham). The immunodetection of proteins was performed using anti-FlgE (1/3000), anti-FliC (1/3000) or anti-VirB8 (1/1000) rabbit polyclonal sera [21], [76], and with anti-Omp89 mAb (A53/10B2) (1/1000), or polyclonal anti-PrlR antibodies (1/1000) [77]. The detection of primary antibodies was performed using donkey anti-rabbit (Amersham) horseradish peroxidase-conjugated secondary antibodies, and visualized using the ECL system (Amersham). The measured molecular masses of FlgE, FliC and VirB8 are 41, 29 and 26,5 kDa, respectively.

### Flow cytometric analysis

Validation of the QS reporter strains was performed as follows. *E. coli* and *B. melitensis* control of QS reporter strains were grown overnight in the conditions described above, and were back-diluted to an OD_600_ of 0.2 in medium supplemented with AHLs at the indicated concentrations. After a 4h- or an 8h-incubation, bacteria were washed in PBS and fixed in 2% paraformaldehyde, pH 7.4, at 37°C for 15min. After an additional wash in PBS, bacteria were used for flow-cytometric analysis of GFP(ASV) production with a FACScalibur using CellQuest software (Becton Dickinson) as previously described [78]. Bacteria were gated according to size and scatter to eliminate debris from analysis. Then, 50,000 individual events were excited with a 488-nm argon-ion laser, and emission light was detected through a 530-nm bandpass filter. The same process of fixation, wash and flow cytometric analysis method was used to follow *B. melitensis* endogenous AHLs activity during *in vitro* growth.

### Cellular infection and immunofluorescence

RAW264.7 murine macrophages were inoculated at a multiplicity of infection of 300:1. Plating serial dilutions of the inocula validated infectious doses. Briefly, bacteria were centrifuged onto cells at 400 *g* for 10 min at 4°C and then incubated 1h at 37°C with 5% CO_2_ atmosphere. Cells were washed twice with medium and then incubated in medium supplemented with 50 µg ml^−1^ gentamicin to kill extracellular bacteria. For bacterial counts, cells were washed twice with PBS and lysed in PBS/0.1% X-100 triton (Sigma). Serial dilutions in PBS were plated onto 2YT media plates, and CFUs were counted after 3 days of incubation at 37°C. For fluorescence microscopy analysis, cells were fixed in 2% paraformaldehyde, pH 7.4, at 37°C for 15min and then processed for immunofluorescence labeling with A76-12G12 anti-LPS monoclonal primary antibody (undiluted hybridoma culture supernatant) and a goat anti-mouse IgG secondary antibody coupled to Texas Red (1/500) (Invitrogen) as previously described [79]. DAPI (4,6-diamidino-2-phenylindole) was incubated along with the secondary antibody to label DNA. Fluorescence analysis was performed with a Nikon i80 microscope. The proportion of GFP(ASV)-positive bacteria after infection by QS reporter strains was determined as follows. At each time p.i., the GFP(ASV) mean fluorescence intensity of individual bacteria was determined as the mean pixel intensity of bacteria observed with the FITC filter by using the Nikon’s imaging software NIS-Elements. We subtracted from these values the green fluorescence background (autofluorescence) that was defined as the mean fluorescence intensity of bacteria bearing the control plasmid plus 2 standard deviations. After infection with the QS reporter strains, bacteria were considered as GFP(ASV)-positive if their fluorescence signal was clearly above the background, i.e. above a threshold of fluorescence intensity arbitrarily fixed to 100.

## Supporting Information

Figure S1 **The quorum sensing reporter system allows** ***in situ*** **semi-quantitative detection of long chain AHLs.** **(A)** Schematic drawings of the QS reporter strain and its control. The plasmid carried by the QS reporter strain contains the divergently transcribed *Plac-lasR* and *PlasB-gfp*(ASV) fusions. The *Plac* drives constitutive expression of *lasR*, whose product induces *gfp*(ASV) by direct binding of the *PlasB* in the presence of long-chain AHLs. The plasmid carried by the control strain does not contain the *Plac-lasR* fusion and is used to control that no endogenous regulator in the strain interferes with the system. Solid lines and dashed lines indicate respectively expected and unexpected interactions, based on the literature. Dotted lines on right panel indicate unknown interactions that are controlled in this study. **(B)** Validation of the specificity and sensitivity of the QS reporter system in *E. coli*. The graphs represent measurement of GFP(ASV) fluorescence intensity in bacteria by flow cytometry (5×10^4^ events acquired) after a 4h-incubation with various AHLs. The control strain incubated with 1 µM of C12-HSL was used as a negative control. The results are representative of two independent experiments.
(TIF)

Figure S2 **Multiple sequence alignment of** ***Brucella*** **AibP with characterized or predicted Ntn hydrolases.** In addition to *B. suis* 1330 AibP (BR_A1089) and *B. melitensis* AibP (BMEII0212 and BMEII0211), AHL-acylases from *Pseudomonas aeruginosa* (PvdQ, QuiP and the putative AHL-acylase PA1893), *Pseudomonas syringae* (HacA and HacB), *Ralstonia eutropha* (predicted AHL-acylase Reut_A1841), *Ralstonia* strain Xj12B (AiiD), *Ralstonia solanacearum* (Aac) and *Streptomyces* strain M664 (AhlM), as well as cephalosporin acylase (CAD) from *Pseudomonas diminuta* and penicillin G acylase (PGA) from *E. coli* were used in the alignment (ClustalW). Stretches of amino acids with no similarity have been collapsed into numbers that occur in the primary sequence. The ‘*’ symbol indicates identical residues, the ‘:’ symbol indicates conserved substitutions, the ‘.’ symbol indicates semi-conserved substitution. Yellow boxes indicate conserved residues of relevance to autoproteolysis and catalysis in characterized Ntn hydrolases.
(TIFF)

Figure S3 **Stronger response of** ***B. melitensis*** **QS reporter strain to AHLs in the absence of** ***aibP*** **.** *B. melitensis* wt and ▵*aibP* QS reporter strains grown to end log phase were incubated separately for 8 hours with C12-HSL (left panel) or 3-oxo-C12-HSL (right panel) (0.1 nM or 1 nM) prior to fixation and analysis of GFP(ASV) fluorescence intensity by flow cytometry (5×10^4^ events acquired). The *B. melitensis* control strain was used as a negative control. Results are representative of at least two independent experiments.
(TIF)

Figure S4 **AHLs activity of** ***B. melitensis*** **QS reporter strains is lower when grown in RPMI-erythritol medium.** *B. melitensis* wt and ▵*aibP* QS reporter strains were grown in 2YT or in RPMI medium supplemented with erythritol (2gl^−1^) for 24h before fixation and fluorescence microscopy analysis in order to determine the percentage of GFP(ASV)-positive bacteria as described in the Material and Methods section.
(TIF)
