# Supplementary figures and images for: Quorum Sensing and Self-Quorum Quenching in the Intracellular Pathogen Brucellamelitensis
Source: PLoS One. 2013 Dec 11;8(12):e82514. doi: 10.1371/journal.pone.0082514 (PMC3859601; doi:10.1371/journal.pone.0082514)

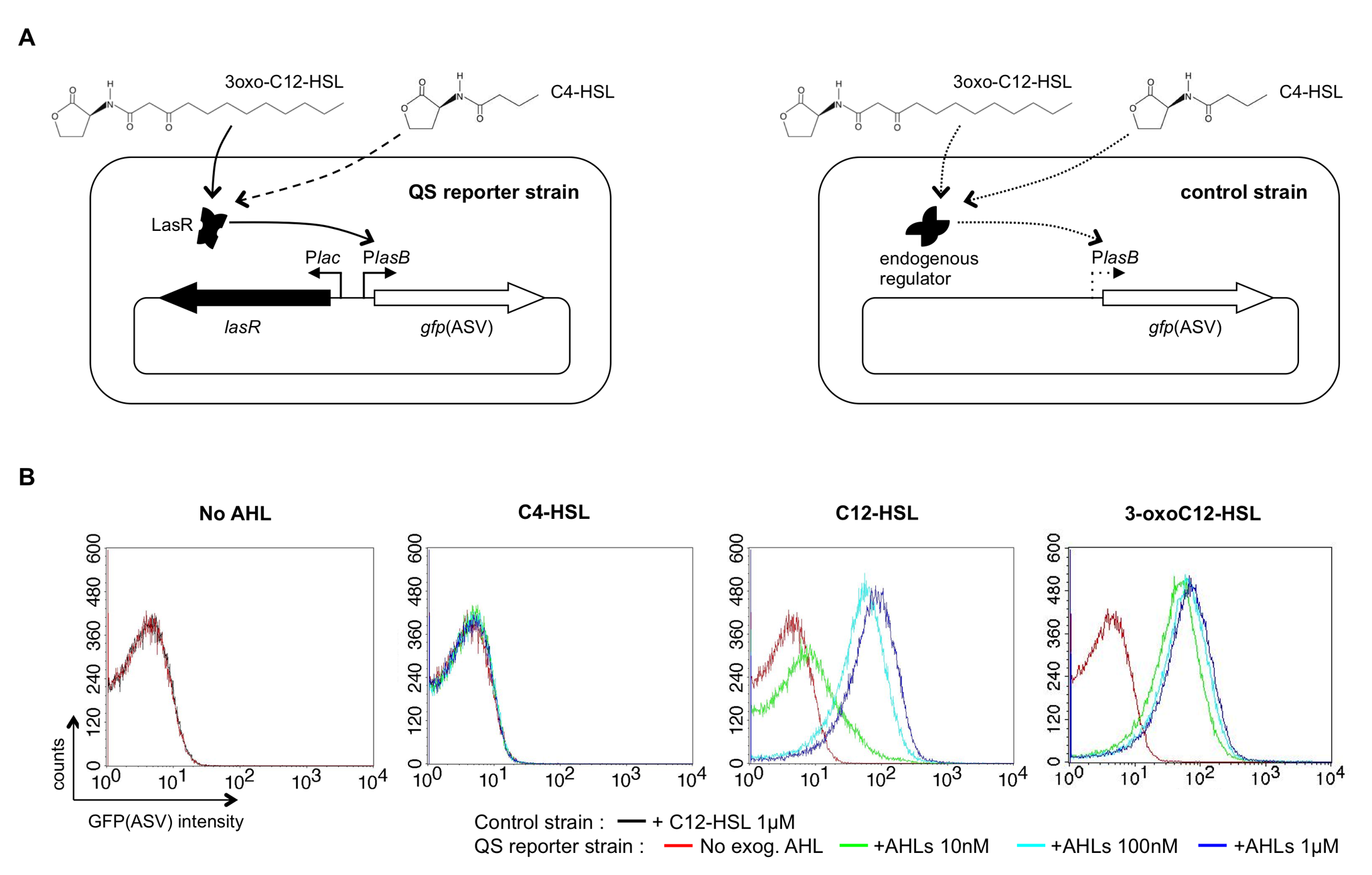

Supplement: Figure S1 — The quorum sensing reporter system allows in situ semi-quantitative detection of long chain AHLs. (A) Schematic drawings of the QS reporter strain and its control. The plasmid carried by the QS reporter strain contains the divergently transcribed Plac-lasR and PlasB-gfp(ASV) fusions. The Plac drives constitutive expression of lasR, whose product induces gfp(ASV) by direct binding of the PlasB in the presence of long-chain AHLs. The plasmid carried by the control strain does not contain the Plac-lasR fusion and is used to control that no endogenous regulator in the strain interferes with the system. Solid lines and dashed lines indicate respectively expected and unexpected interactions, based on the literature. Dotted lines on right panel indicate unknown interactions that are controlled in this study. (B) Validation of the specificity and sensitivity of the QS reporter system in E. coli. The graphs represent measurement of GFP(ASV) fluorescence intensity in bacteria by flow cytometry (5×10^4^ events acquired) after a 4h-incubation with various AHLs. The control strain incubated with 1 µM of C12-HSL was used as a negative control. The results are representative of two independent experiments. (TIF) [file pone.0082514.s001.tif]

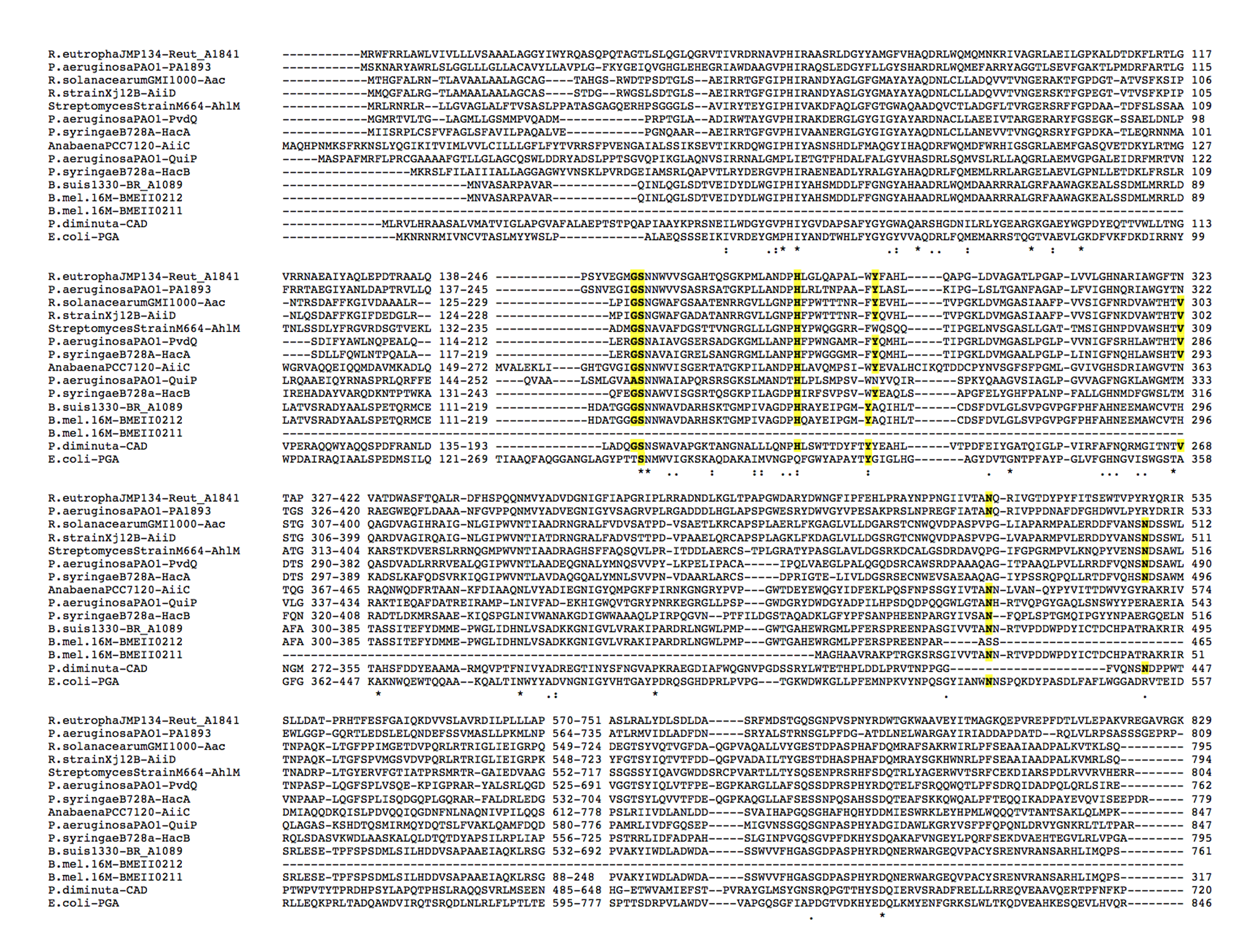

Supplement: Figure S2 — Multiple sequence alignment of Brucella AibP with characterized or predicted Ntn hydrolases. In addition to B. suis 1330 AibP (BR_A1089) and B. melitensis AibP (BMEII0212 and BMEII0211), AHL-acylases from Pseudomonas aeruginosa (PvdQ, QuiP and the putative AHL-acylase PA1893), Pseudomonas syringae (HacA and HacB), Ralstonia eutropha (predicted AHL-acylase Reut_A1841), Ralstonia strain Xj12B (AiiD), Ralstonia solanacearum (Aac) and Streptomyces strain M664 (AhlM), as well as cephalosporin acylase (CAD) from Pseudomonas diminuta and penicillin G acylase (PGA) from E. coli were used in the alignment (ClustalW). Stretches of amino acids with no similarity have been collapsed into numbers that occur in the primary sequence. The ‘*’ symbol indicates identical residues, the ‘:’ symbol indicates conserved substitutions, the ‘.’ symbol indicates semi-conserved substitution. Yellow boxes indicate conserved residues of relevance to autoproteolysis and catalysis in characterized Ntn hydrolases. (TIFF) [file pone.0082514.s002.tiff]

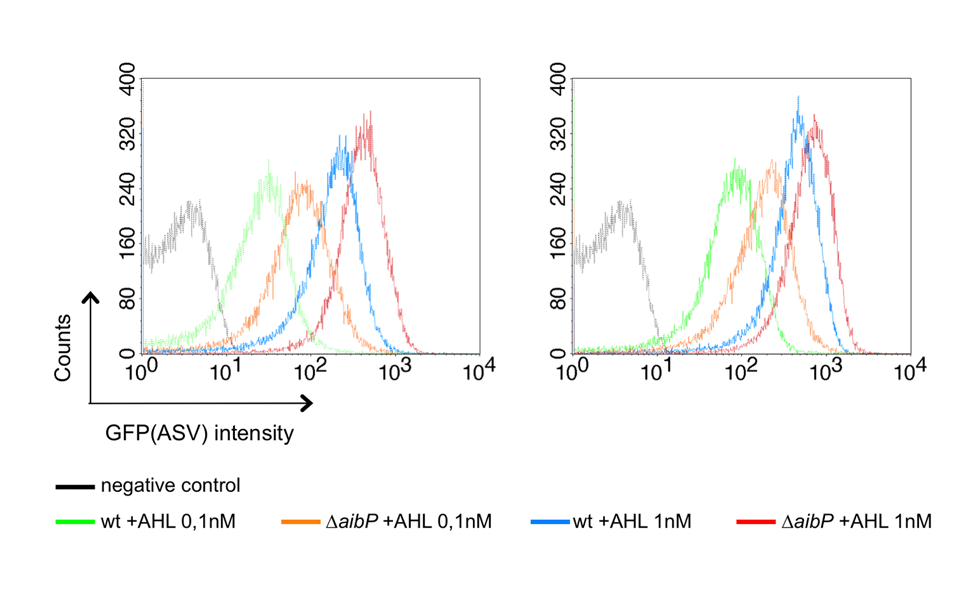

Supplement: Figure S3 — Stronger response of B. melitensis QS reporter strain to AHLs in the absence of aibP . B. melitensis wt and ▵aibP QS reporter strains grown to end log phase were incubated separately for 8 hours with C12-HSL (left panel) or 3-oxo-C12-HSL (right panel) (0.1 nM or 1 nM) prior to fixation and analysis of GFP(ASV) fluorescence intensity by flow cytometry (5×10^4^ events acquired). The B. melitensis control strain was used as a negative control. Results are representative of at least two independent experiments. (TIF) [file pone.0082514.s003.tif]

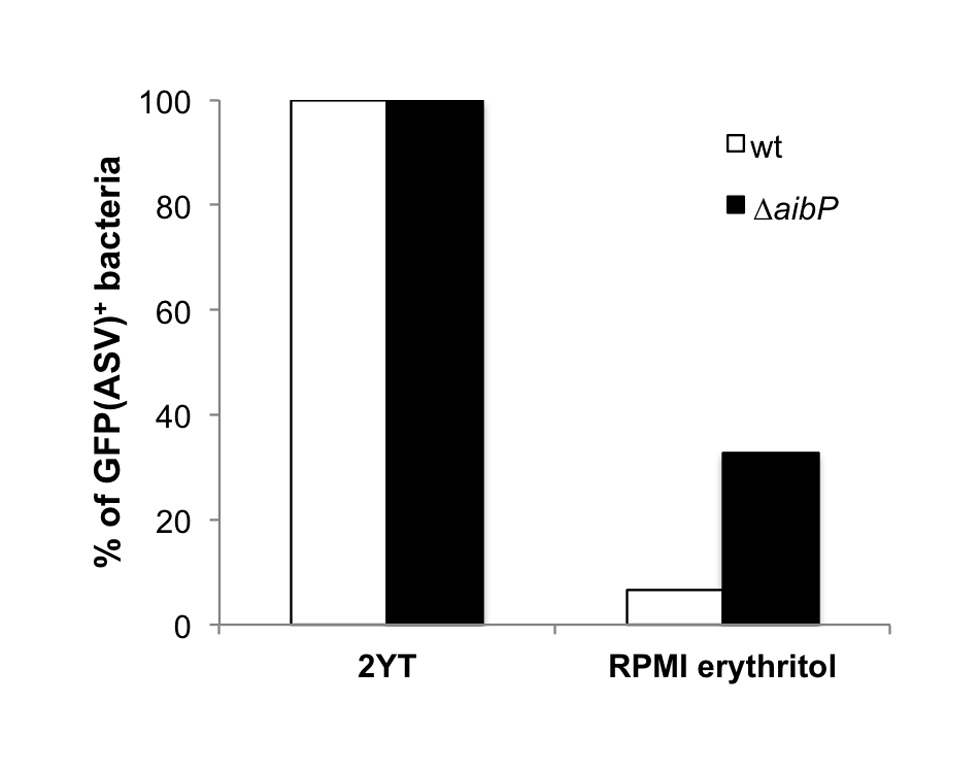

Supplement: Figure S4 — AHLs activity of B. melitensis QS reporter strains is lower when grown in RPMI-erythritol medium. B. melitensis wt and ▵aibP QS reporter strains were grown in 2YT or in RPMI medium supplemented with erythritol (2gl−1) for 24h before fixation and fluorescence microscopy analysis in order to determine the percentage of GFP(ASV)-positive bacteria as described in the Material and Methods section. (TIF) [file pone.0082514.s004.tif]
